# Exploring the Structural Insights of Thermostable *Geobacillus* esterases by Computational Characterization

**DOI:** 10.1021/acsomega.4c03818

**Published:** 2024-07-22

**Authors:** Yusuf Sürmeli, Naciye Durmuş, Gülşah Şanlı-Mohamed

**Affiliations:** † Department of Agricultural Biotechnology, Tekirdağ Namık Kemal University, 59030 Tekirdağ, Turkey; ‡ Department of Molecular Biology and Genetics, İstanbul Technical University, 34485 İstanbul, Turkey; § Department of Chemistry, İzmir Institute of Technology, 35430 İzmir, Turkey

## Abstract

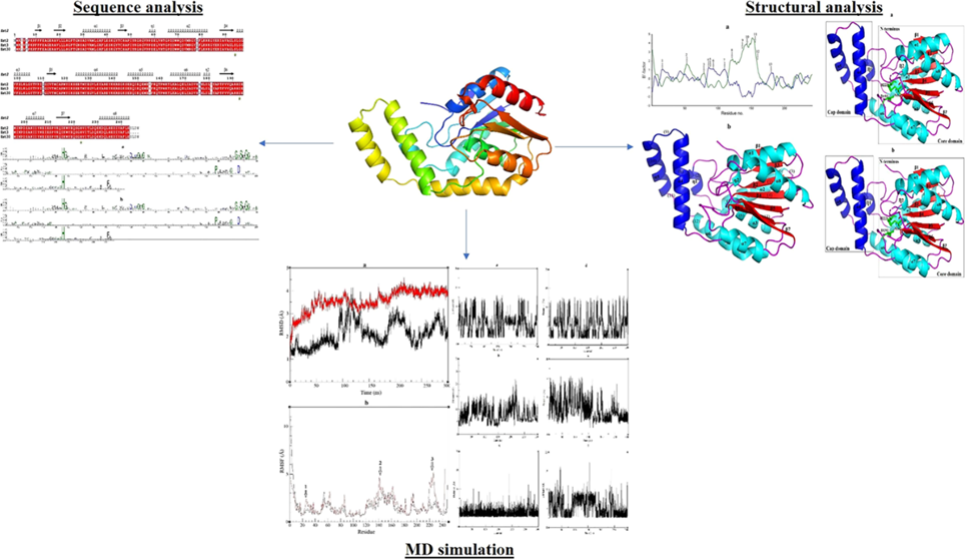

This study conducted an *in silico* analysis of two biochemically characterized thermostable esterases, Est2 and Est3, from *Geobacillus* strains. To achieve this, the amino acid sequences of Est2 and Est3 were examined to assess their biophysicochemical properties, evolutionary connections, and sequence similarities. Three-dimensional models were constructed and validated through diverse bioinformatics tools. Molecular dynamics (MD) simulation was employed on a *p*NP-C2 ligand to explore interactions between enzymes and ligand. Biophysicochemical property analysis indicated that aliphatic indices and theoretical *T*_m_ values of enzymes were between 82–83 and 55–65 °C, respectively. Molecular phylogeny placed Est2 and Est3 within Family XIII, alongside other *Geobacillus* esterases. DeepMSA2 revealed that Est2, Est3, and homologous sequences shared 12 conserved residues in their core domain (L39, D50, G53, G55, S57, G92, S94, G96, P108, P184, D193, and H223). BANΔIT analysis indicated that Est2 and Est3 had a significantly more rigid cap domain compared to Est30. Salt bridge analysis revealed that E150–R136, E124–K165, E137–R141, and E154–K157 salt bridges made Est2 and Est3 more stable compared to Est30. MD simulation indicated that Est3 exhibited greater fluctuations in the N-terminal region including conserved F25, cap domain, and C-terminal region, notably including H223, suggesting that these regions might influence esterase catalysis. The common residues in the ligand-binding sites of Est2–Est3 were determined as F25 and L167. The analysis of root mean square fluctuation (RMSF) revealed that region 1, encompassing F25 within the β2–α1 loop of Est3, exhibited higher fluctuations compared to those of Est2. Overall, this study might provide valuable insights for future investigations aimed at improving esterase thermostability and catalytic efficiency, critical industrial traits, through targeted amino acid modifications within the N-terminal region, cap domain, and C-terminal region using rational protein engineering techniques.

## 1 Introduction

Esterases, classified within the lipolytic hydrolase family, play a role in hydrolyzing and forming ester bonds.^1^ These enzymes are involved in various reactions such as esterification, transesterification, and interesterification.^2^ Their impact on reversible reactions is influenced by thermodynamics. In the organic phase, esterases facilitate ester formation, while in the aqueous phase, they catalyze ester hydrolysis. Notably, esterases differ from lipases based on substrate specificity and surface activation, with esterases targeting shorter-chain carboxylic acids (C-12 or less), whereas lipases act on longer-chain triglycerides (exceeding C-12), which are insoluble.^3−5^ The classification of microbial lipases/esterases relies on the amino acid sequence, resulting in the identification of eight families (I–VIII).^6^ Over time, this classification has been expanded with the addition of new families, reaching up to XVIII. Some of these families have been discovered more recently through metagenomic approaches.^7,8^

Esterases find diverse applications in biotechnology, serving as additives in laundry detergents and playing a role as stereospecific biocatalysts in the production of pharmaceuticals.^9^ These enzymes vary in their substrate specificity, allowing for their utilization in a range of medical applications.^10^ However, many industrial processes involve harsh conditions that can deactivate these enzymes. Therefore, there is a high demand for novel esterases exhibiting improved catalytic efficiency and specific properties suitable for particular reaction conditions.^11^ The thermostability of an enzyme is a crucial characteristic, especially considering its proposed applications in biotechnology.^12^

The organisms belonging to the *Geobacillus* genus are mandatory thermophiles and exhibit a Gram-positive nature. They possess the capability to thrive within the temperature range of 45–80 °C.^13,14^*Geobacillus* organisms produce enzymes with notable thermostability, demonstrating resilience to challenging conditions like detergents, organic solvents, pH fluctuations, and chemical denaturants.^15−22^ There is currently a substantial endeavor to discover thermostable esterases from *Geobacillus* species found in diverse geographical locations.^23,24^ The geothermal waters in Balçova, located in zmir, Turkey, serve as habitats for thermophilic *Geobacillus* species, which act as reservoirs for a range of thermostable enzymes. Various thermostable enzymes such as α-l-arabinofuranosidase, xylanase, protease, α-amylase, esterases, and lipases have been effectively generated and characterized from this specific geographic area.^15−19,25,26^ Tekedar and Şanlı-Mohamed (2011) biochemically characterized three esterases (Est1, Est2, and Est3) of *Geobacillus* sp. from geothermal waters in Balçova, located in İzmir, Turkey, which exhibited optimal activity at a temperature of 65 °C and a pH range of 9.5–10.0.^17^ These esterases preferentially catalyzed the formation of the *p*NP-C2 ligand. In this research, two biochemically characterized thermally stable esterases (Est2 and Est3) originating from *Geobacillus* sp., isolated from the Balçova geothermal area in zmir, Turkey, were subjected to in silico analysis at various levels including phylogenetics, sequence, structure, and enzyme–substrate interactions.

## 2 Materials and Methods

Two experimentally characterized esterases of *Geobacillus* strains (Est2 and Est3) from Balçova geothermal site^17^ were used for the *in silico* analysis. For this purpose, Est2 and Est3 amino acid sequences were obtained from the NCBI database FN597622 and FN597623 accession numbers, respectively.

### 2.1 Biophysicochemical Characteristics

The biophysicochemical properties of Est2 and Est3 were determined via ProtPram tool^27^ and *T*_m_ predictor program.^28^ For this purpose, the theoretical isoelectric point (pI), aliphatic index, instability index, molecular mass, aa length, and grand average of hydropathicity (GRAVY) were predicted using the enzyme amino acid sequences by ProtPram tool. In addition, a range of *T*_m_ values of the enzymes were estimated by *T*_m_ predictor online program.

### 2.2 Phylogenetic and Amino Acid Sequence Analyses

The molecular phylogeny analysis of the esterases with the other characterized esterases from different esterase families were performed by MEGA11 software. To do this, the analysis took place by using the Unweighted Pair Group Method with Arithmetic Mean (UPGMA) statistical method with 1000 bootstrap replications.^29^ The phylogenetic tree was monitored by the Interactive Tree of Life (iTOL) tool.^30^ In addition to these, the amino acid sequence alignment of the esterases was analyzed via Clustal Omega^31^ and monitored by the ESPript program.^32^ In addition, DeepMSA2 (Deep Multiple Sequence Alignment 2), an MSA generation pipeline was used to align each thermostable enzyme (Est2 and Est3) with the homologous esterases in the Uniclust30, Uniref90, BFD, MGnify, and IMG/M databases.^33^

### 2.3 Homology Modeling

Three-dimensional (3D) homology models of the esterases were built via ProMod3 software in the SWISS-MODEL server.^34−37^ The models were cross-validated using various bioinformatics tools. Accordingly, the ProSA server was used to assess the local and global quality of the models via the determination of their *Z* scores.^38^ In addition, ProCheck was utilized to investigate the stereochemical qualities and dihedral angles and to generate their Ramachandran plots.^39^ The Stride was also used to predict the secondary structures of the esterase models.^40^ The Verify3D was utilized to predict the compatibility between the 3D structures and their amino acid sequences, in reference to known structures.^41^ Alphafold models of Est2 and Est3 were obtained by AlphaFold Colab.^42^

*B*′-factor analysis of the 3D models of the esterases was estimated via BANΔIT.^43^ To do this, the .pdb files of the models were used for the determination of the *B*′-factor values per residue of each enzyme model. A lower *B*′-factor was an indicator of a more rigid region and thereby higher stability.

### 2.4 Molecular Dynamics (MD) Simulation

Molecular dynamics (MD) simulations were employed to explore the interactions between enzymes and the *p*NP-C2 ligand in the case of Est2 and Est3 throughout 300 ns. The ligand was positioned within the active site of the protein structures, and a full MD simulation was conducted for the protein–ligand complex to provide a deeper understanding of the dynamic structural changes and interactions governing the active site. MD simulations were performed using Amber 16 software, employing the ff14SB and tip3p force fields. The ligand structure was obtained and prepared, which involved tasks such as adding hydrogen atoms, assigning charges, and generating parameter and topology files using an antechamber and the gaff force field. An input script for the leap program was generated to load both the protein and the ligand, establish the system, and immerse it within a water box utilizing the tip3p water model. Energy minimization was carried out to address steric conflicts and achieve system relaxation. A Langevin thermostat was employed to regulate the temperature while initiating the MD simulation in the NVT ensemble with temperature settings at 338 K and a time step of 0.002 ps. Snapshots of the trajectory were saved at regular intervals for subsequent analysis. After the MD simulation, an analysis of the trajectory was conducted to gain an understanding of the interactions between the protein and ligand as well as to assess any structural modifications and pertinent attributes. The trajectories and molecular structures were visualized using PyMOL. Root mean square deviation (RMSD), root mean square fluctuation (RMSF), nonparametric clustering, and distance analyses were performed to elucidate the dynamics of Est2 and Est3. During the MD simulation, the RMSF values were calculated by assessing the variance in the position of individual residues from their mean structure. Additionally, distances between particular atoms were determined through simulations utilizing cpptraj. The RMSD values for the simulation were determined by comparing the atomic coordinates of each frame with a reference structure by using cpptraj. Distance measurements and algorithms for clustering are available in a wide variety, and certain combinations are effective for particular systems. We examined three clustering algorithms (*k*-means, dbscan, and hierarchical agglomerative) that are available in cpptraj to find the optimal technique that might yield the most information about our system.

### 2.5 Data Visualization

The figures were visualized by GraphPad Prism version 6.00 for Windows (GraphPad Software, La Jolla, CA) (www.graphpad.com), MEGA11 software, and PyMOL Molecular Graphics System, Version 2.0 (Schrödinger, LLC).

## 3 Results

### 3.1 Biophysicochemical Features of Amino Acid Sequences of Esterases

The biophysicochemical features including the theoretical melting temperature (*T*_m_) by the *T*_m_ predictor program, and theoretical pI, GRAVY, aliphatic index, aa length, and molecular mass by ProtParam tool were determined for each thermostable esterase (Est2 and Est3) and reference esterase from *Geobacillus stearothermophilus* (Est30). The results showed that all of the features of the three thermostable esterases were similar to each other and had subtle differences. Accordingly, Est2, Est3, and Est30 possessed a *T*_m_ of 55–65 °C. In line with this, the aliphatic index of the esterases was similar to each other and was found around 82–83. Also, the theoretical pI of Est2 and Est3 was found as about 5.25, whereas the pI of Est30 was found as 5.03 (Table 1).

**Table 1:** Biophysicochemical Features (Theoretical pI, GRAVY, Aliphatic Index, aa Length, Molecular Mass, and Theoretical *T*_m_) of Thermostable Esterases (Est2, Est3, and Est30)

no.	protein ID	protein name	organism	theoretical pI	GRAVY	aliphatic index	instability index	aa length	molecular mass (kDa)	theoretical *T*_m_ (°C)
1	FN597622	Est2	*Geobacillus* sp.	5.24	–0.366	82.47	41.07	247	28.34	55–65
2	FN597623	Est3	*Geobacillus* sp.	5.25	–0.363	82.22	40.66	243	27.84	55–65
3	1TQH	Est30	*G. stearothermophilus*	5.03	–0.383	82.87	43.15	247	28.38	55–65

### 3.2 Determination of the Molecular Phylogeny of Thermostable Esterases

Arpigny and Jaeger initially categorized bacterial lipolytic enzymes into families I–VIII based on their amino acid sequences and substrate preferences.^6^ However, with the identification of additional lipolytic enzymes, the classification system was expanded to encompass a total of 19 distinct families.^44^ In this work, the molecular phylogeny of two thermostable esterases from *Geobacillus* sp. was determined by the MEGA11 program using the Unweighted Pair Group Method with Arithmetic Mean (UPGMA) statistical method and 1000 bootstrap replications. This analysis indicated that Est2 and Est3 belonged to Family XIII, together with the *G. stearothermophilus* esterases as *Gs*Est (PDB: 1R1D) and Est30 (PDB: 1TQH) (Figure S1). Esterases belonging to this particular family, weighing around 28 kDa, were identified in *Bacillus* and *Geobacillus* genera, which are closely related in evolution and belong to the endospore-forming Gram-positive bacteria.^45^

### 3.3 Multiple Sequence Alignment of Amino Acid Sequences of Thermostable Esterases

**Figure 1:**
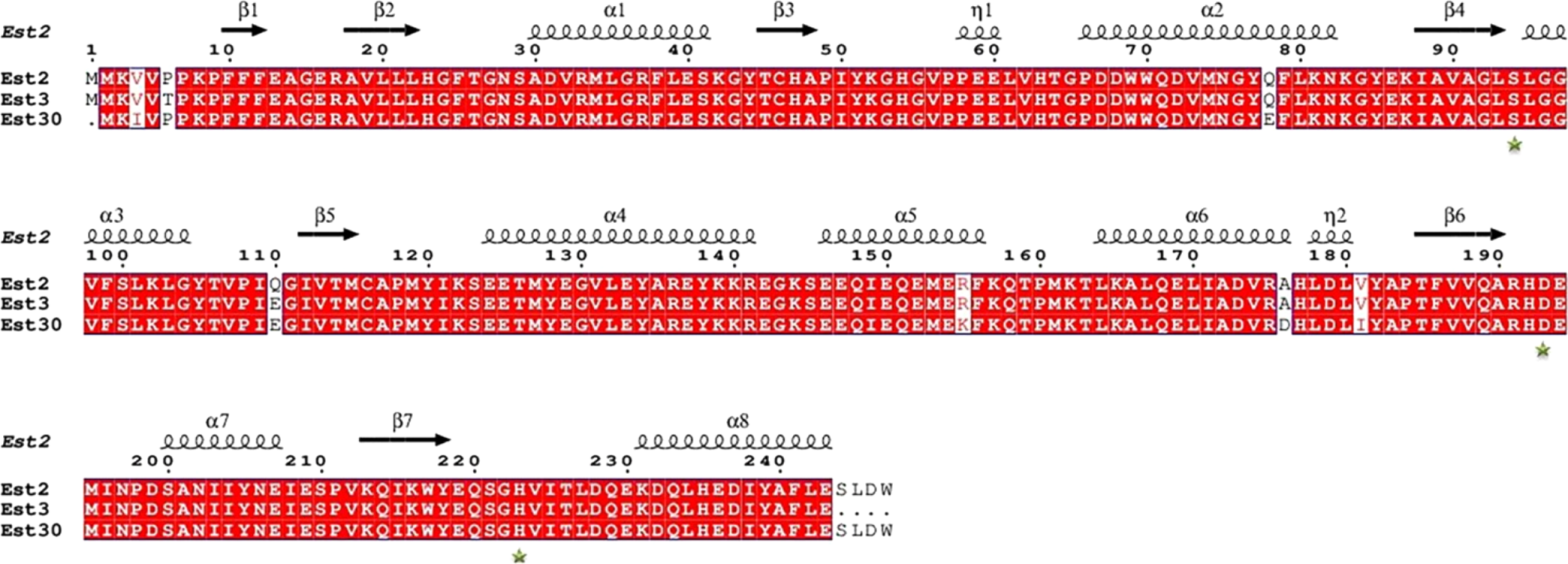
Multiple sequence alignment of Est2, Est3, and Est30 from *G. stearothermophilus*. The red background in the figure serves to highlight residues that are strictly conserved, while conservatively substituted residues are marked within boxes. The aligned sequences of Est2 revealed the secondary structural elements, such as α-helices (α) and β-strands (β), situated above them. The conserved catalytic triad, specifically S94, D193, and H223, were depicted by green asterisks. The figure was generated by ESPript.^32^

The ESPript program played a significant role as a bioinformatics tool in elucidating the fundamental features within protein structures.^32^ In the present work, the amino acid sequence alignment of Est2 and Est3, compared to Est30, was carried out by Clustal Omega and monitored by the ESPript program. The analysis results indicated that Est2 and Est3 were highly similar to each other and Est30, possessing small differences throughout the sequences. Regarding this, Est2 had P6 and Q110, whereas Est3 possessed T6 and E110 at the corresponding positions. In addition, the extra four amino acids (SLDW) in the C-terminal of Est2 were present, relative to Est3. The results also indicated that Est2 and Est3 had five differences (I4, Q78, R155, A176, and V181, in the N-terminal site, α2, α5, α6, and η2–β6 loop, respectively), relative to the characterized Est30 including V4, E78, K155, D176, and I181 as corresponding amino acids (Figure 1).

DeepMSA2 (Deep Multiple Sequence Alignment 2) served as a pipeline for generating multiple sequence alignments (MSA) for both single and multichain proteins. It facilitated the construction of MSAs with homologous sequences extracted from genomic and metagenome sequence databases.^33^ In this study, DeepMSA2 was used to align each thermostable enzyme (Est2 and Est3) with the homologous esterases in the Uniclust30, Uniref90, BFD, MGnify, and IMG/M databases to determine the evolutionarily conserved residues in two thermostable esterases. In this analysis, the alignment depths (Nf) of Est2 and Est3 amino acid sequences were found to be 1593.6 and 2408.97, whereas the aligned number of sequences were 30,094 and 43,657, respectively. In DeepMSA2 results, the larger a letter is, the more conserved it is. The results showed that the conserved residues of L39, D50, G53, G55, S57, G92, S94, G96, P108, P184, D193, and H223 in homologous sequences were commonly found in two thermostable esterases (Figure S2). MSA results showed that L39 in α1 helix, D50, G53, G55, S57 in β3–η1 loop, G92 in β4 strand, S94 in β4–α3 loop, G96 in α3 helix, P108 in α3–β5 loop, P184 in η2–β6 loop, D193 in β6–α7 loop, and H223 in β7–α8 loop were placed in Est2 and Est3 (Figure 1). Among these, the catalytic triad (S94, D193, and H223) was present as conserved residues. On the other hand, the conserved residues of A104, Y109, L186, and G190 in homologous sequences were substituted by G104, I109, F186, and A190 in Est2 and Est3 (Figure S2). MSA analysis indicated that G104, I109, F186, and A190 were placed in the α3 helix, α3–β5 loop, and β6 strand of Est2 and Est3, respectively (Figure 1).

### 3.4 Prediction of the Three-Dimensional Structure of Esterases

The predicted 3D structures of two thermostable esterases were built by a SWISS-MODEL homology modeling online server. The best template was selected according to the percentage of amino acid sequence identity, coverage, and the global model quality estimate (GMQE) value. The most suitable template was determined as the structure of a carboxylesterase (PDB: 1R1D.1A) from *G. stearothermophilus* (*Gs*Est). The homology modeling indicated that the percent identity of the amino acid sequences of Est2 and Est3 was found as 99.19 and 99.18%, respectively. QMEANDisco and GMQE scores showed that the predicted models of the esterases were of high quality (Table 2).

**Table 2:** SWISS-MODEL Scores (Sequence Identity, Coverage, GMQE, and QMEANDisCo) of Homology Models of Thermostable Esterases (Est2 and Est3)

protein name	template	sequence identity (%)	coverage (%)	GMQE	QMEANDisCo
Est2	1R1D	99.19	100	0.94	0.90 ± 0.05
Est3	1R1D	99.18	100	0.94	0.90 ± 0.05

The validation of the anticipated model configurations for Est2 and Est3 was conducted through diverse bioinformatics tools, as outlined in Figures S3 and S4. The three-dimensional forecasted model structures for Est2 and Est3, generated with SWISS-MODEL, were displayed in Figures S3a and S4a, respectively. The structural comparison between the homology model and the alphafold model revealed a high degree of similarity in their structural patterns, indicating strong compatibility between them (Figures S3b and S4b). The same results were obtained from an alignment of the 3D homology model and its template (Figures S3c and S4c). Also, while Est2 and Est3 each had only one outlier residue, a substantial majority of the residues, accounting for 93.3 and 93.7%, respectively, were located within the most favored regions. It can be acknowledged that these models were of high quality, given that they contained over 90% of residues in the most favored regions (Figures S3d and S4d). Additionally, Figures S3e and S4e depict the secondary structure analysis of the forecasted models conducted using Stride. In addition, QMEAN scores of −0.05 for Est2 and −0.08 for Est3 further confirm the high quality and native-like nature of the predicted models, as these scores approach zero (Figures S3f and S4f). The ProSA server was used to assess the overall quality of the homology models for Est2 and Est3, relying on *Z* scores. The examination revealed that the homology models for Est2 and Est3 obtained *Z* scores of −9.93 and −10, respectively, falling below the negative energy cutoff, which signifies their high quality. The *Z* score is calculated based on the scores of other structures of comparable size obtained through experimental techniques such as NMR and X-ray (Figures S3g and S4g).

### 3.5 Structural Investigation of Thermostable Esterases

**Figure 2:**
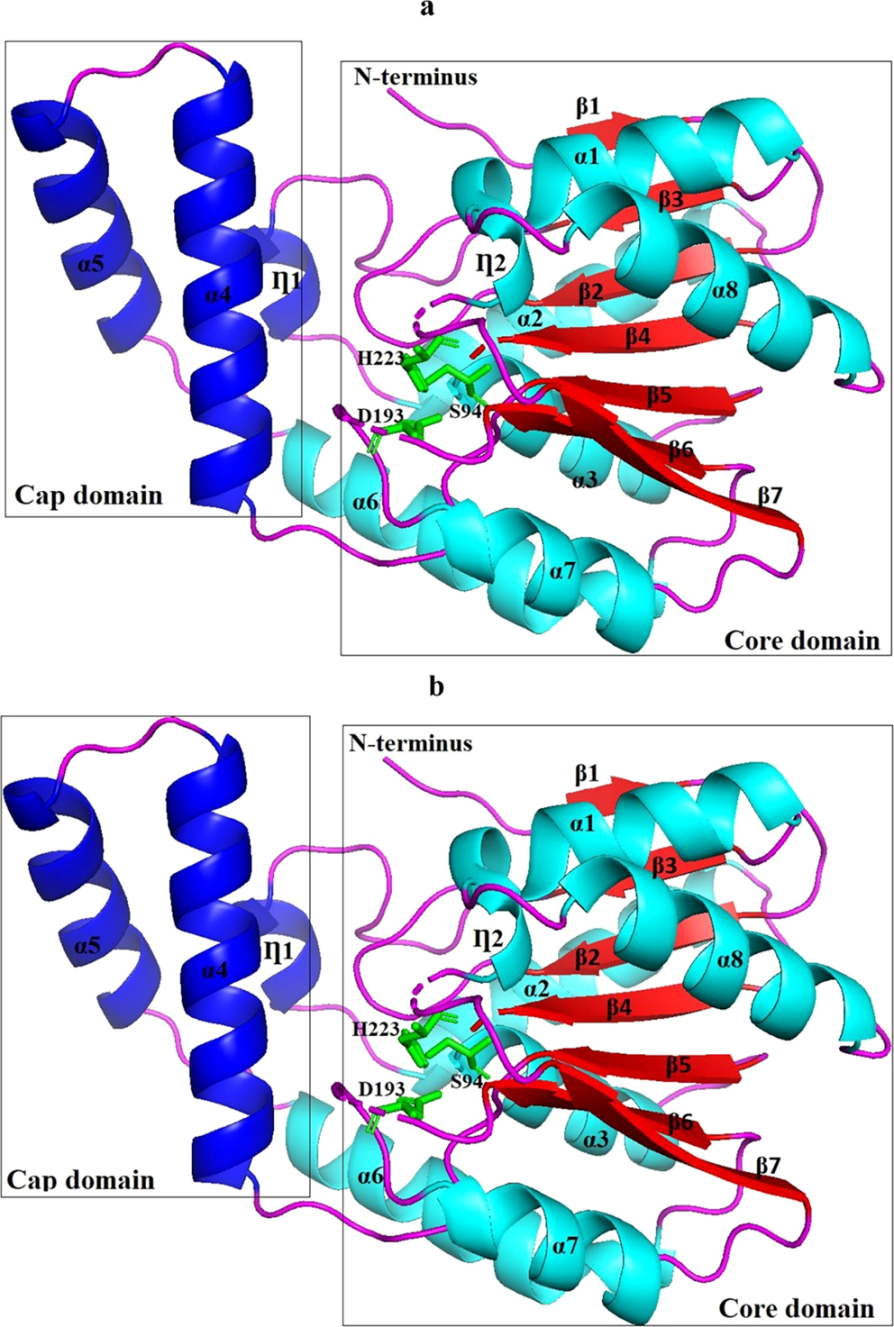
α/β-Hydrolase fold core domain and cap domain in two thermostable esterases Est2 (a) and Est3 (b). The catalytic triad residues were depicted by a green color, and the cap domain was represented by a blue color.

Family XIII, which is one of the greatest classes of structurally linked proteins,^46^ originated with the identification of the esterase Est30 from *G. stearothermophilus*.^47,48^ The discovery involved a 3D structural analysis, unveiling a distinctive topological structure comprising a three-helix cap domain and an α/β-hydrolase fold domain.^49^ In this study, the two thermostable esterases were structurally investigated and compared. This investigation revealed the preservation of two distinct domains (cap domain and α/β-hydrolase fold core domain) in two thermostable esterases (Est2 and Est3). Accordingly, the cap domain consisted of two large and one small α-helices (α4, α5, and η1), while the core domain comprised seven β-strands (β1–β7), eight α-helices (α1–α8), and a short helix (η2) in Est2 and Est3 (Figure 2). The structural analysis also indicated that the β1 strand was antiparallel to the other β strands in the esterases. In this study, the esterases had catalytic triad residues as Ser94 in the α3–β4 loop, Asp193 in the α7–β6 loop, and His223 in the α8–β7 loop (Figure 2).

### 3.6 Investigation of Thermostability of Esterases

**Figure 3:**
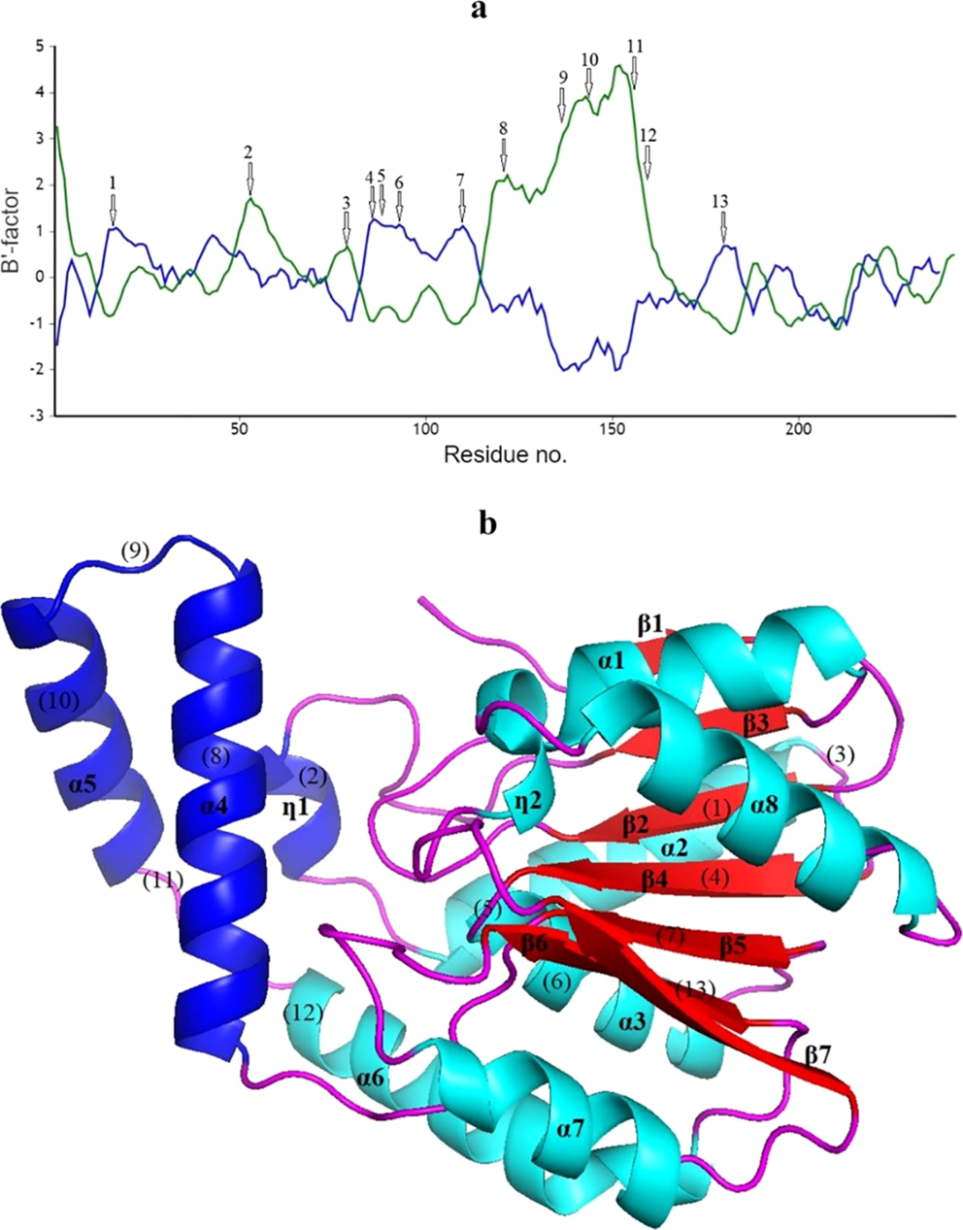
*B*′-factor value distribution of the structurally aligned Est2–Est3, aligned with Est30 (a) and the presentation of differences on the Est2–Est3 aligned structure (b). In the *B*′-factor distribution graph, the blue line refers to Est2–Est3, and the green line depicts Est30.

BANΔIT methodology was performed to assess the rigid and flexible regions of the aligned structures of Est2 and Est3, which exhibited the same normalized temperature factor *B* (*B*′-factor) distribution graph compared to Est30. The analysis revealed noticeable distinctions in the *B*′-factor distributions between aligned Est2–Est3 and that of Est30. Based on these findings, regions 8–11 demonstrated a notably more rigid structure in Est2 and Est3 compared to Est30 (Figure 3a). Among these, regions 8, 9, 10, and 11 were placed in the α4 helix, α4–α5 loop, α5 loop, and α5–α6 loop, respectively, and they were located at the cap domain (Figure 3b).

**Figure 4:**
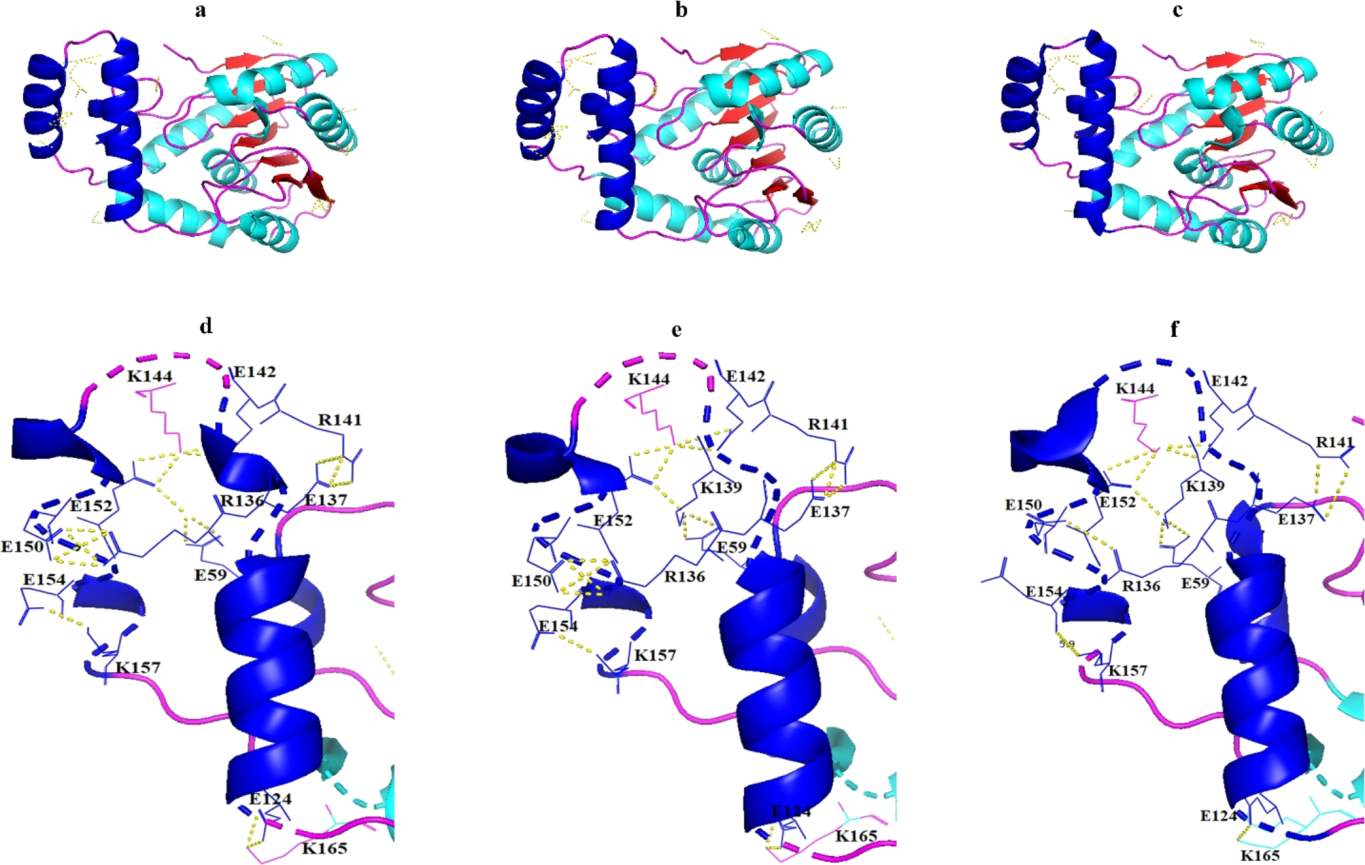
Display of the total salt bridge network on Est2 (a), Est3 (b), and Est30 (c) and the salt bridge network on the cap domain of Est2 (d), Est3 (e), and Est30 (f). The salt bridge between negatively and positively charged residues was depicted by a yellow dashed line. Only the E154–K157 pair in Est30 was indicated to have a distance of 5.9 Å, emphasizing that it is too far apart to form a salt bridge.

In this work, the salt bridges in Est2, Est3, and Est30 were determined. The results showed that each of Est2 and Est3 possessed a total of 28 salt bridges, whereas Est30 had 21 salt bridges. Across all three enzymes, salt bridges were established involving pairs E142–K144, E152–K144, E150–R136, E124–K165, and E137–R141. Notably, Est2 and Est3 exhibited a greater occurrence of salt bridges between E150–R136, E124–K165, and E137–R141 compared to Est30. Moreover, both Est2 and Est3 had an additional salt bridge, E154–K157, which was not present in Est30 because there was too much distance between them to form a salt bridge (5.9 Å) (Figure 4).

### 3.7 MD Simulation

**Figure 5:**
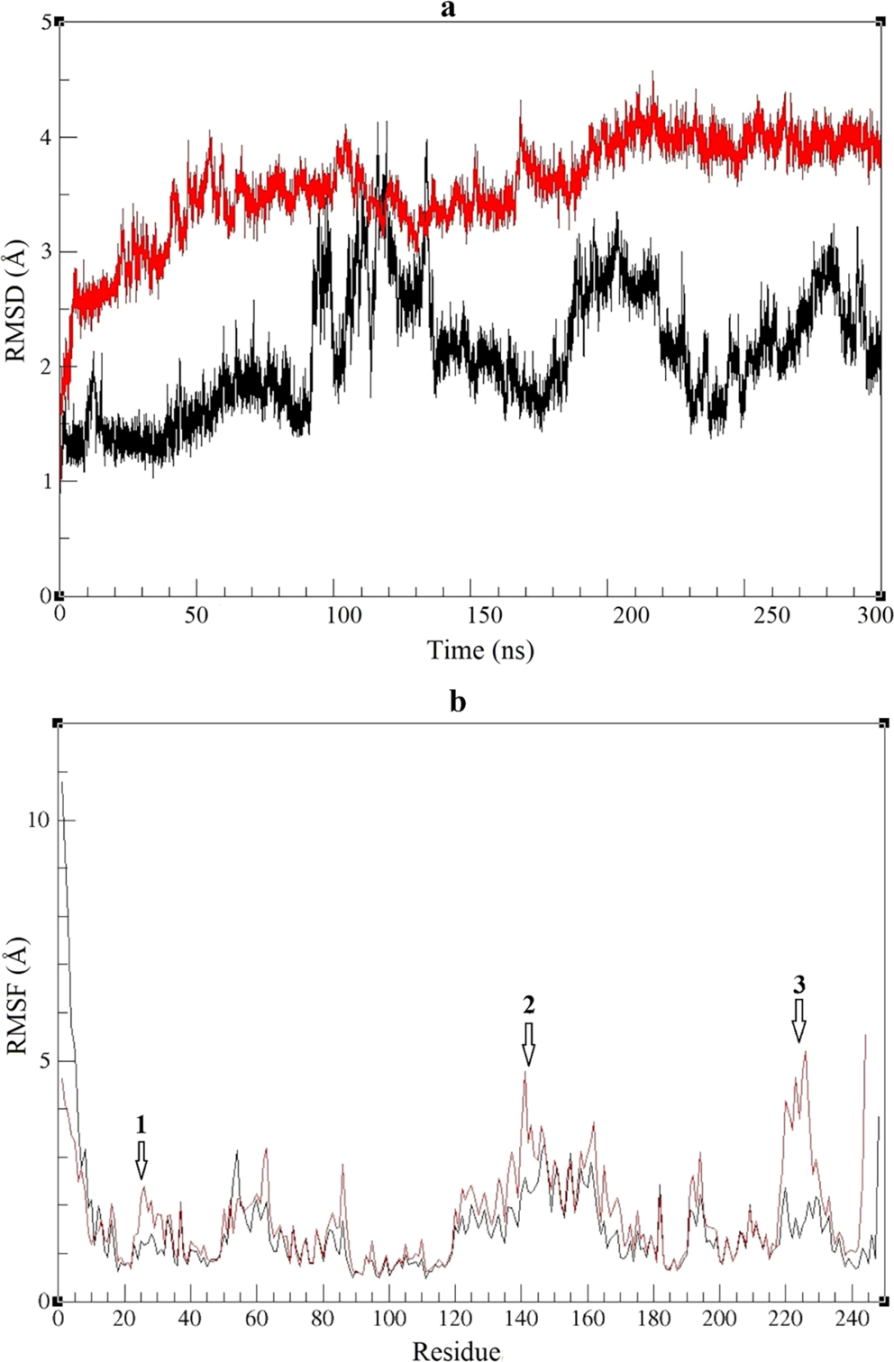
RMSD (a) and RMSF (b) profiles during the molecular dynamics simulation of two thermostable esterases at 338 K over a duration of 300 ns for Est2 (black line) and Est3 (red line).

MD simulation serves as an effective method for establishing the connection between the protein structure and stability, exhibiting notable consistency with relevant experimental data.^50,51^ In this work, MD simulations were used to reveal the important residues interacting with the *p*NP-C2 ligand throughout 300 ns. It has been reported that enzymes within Family XIII exhibit a distinct preference for hydrolyzing short-chain ester substrates (e.g., *p*NP-C2).^49^ Est2 and Est3 acted on the ligand (*p*NP-C2) at a high effective level, compared to the other substrates.^17^ Thus, the *p*NP-C2 ligand was selected for the MD simulation. In the course of this analysis, RMSD, RMSF, nonparametric clustering, and distance analyses were performed to elucidate the dynamics of Est2 and Est3 over time at a temperature of 338 K (65 °C) (Figures 5, S5, and S6), which is the optimum temperature of Est2 and Est3.^17^

RMSD graph was created to illustrate the deviations in the overall structures of Est2 and Est3. The enzyme structures reached equilibrium within time intervals of 50–300 ns, at 343 K for two thermostable esterases (Figure 5a). The RMSF values of Est2 and Est3 were determined in the MD simulation by assessing the variance of each residue’s position relative to its mean structure. When analyzing the RMSF graph, it became evident that the residue fluctuation displayed a comparable pattern for both Est2 and Est3. However, it was established that Est3 exhibited increased significant fluctuations in amino acid movements across three regions, compared to Est2: region 1 included residues 20–30 toward the N-terminal region; region 2 pertained to the location of amino acids 140–145, corresponding to the α4–α5 loop within the cap domain; region 3 encompassed amino acids 220–230 and corresponded to the β7–α8 loop, which included H223 of the C-terminal region of the catalytic domain (Figure 5b).

Nonparametric clustering analysis of MD simulation of Est2 and Est3 was carried out for the determination of the most populated cluster by three different algorithms (*k*-means, dbscan, and hierarchical agglomerative) to determine the optimal method that might yield the most information about our system. The DBI and psF values are metrics of clustering quality; low values of DBI and high values of pSF indicate better results.^52^ As a result, the hierarchical agglomerative algorithm was found as the optimal technique for the clustering of Est2 and Est3 (Table 3).

**Table 3:** Application of Three Different Clustering Algorithms (*k*-Means, dbscan, and Hierarchical Agglomerative) in the Determination of the Optimal Clustering Method for the MD Simulation of Thermostable Est2 and Est3

enzymes	algorithm	DBI	pSF	SSR/SST
Est2	dbscan	1.748931	664.2	0.29
	*k*-means	2.147149	8974.4	0.57
	hierarchical agglomerative	1.871804	7890.4	0.54
Est3	dbscan	1.521462	4123.1	0.65
	*k-*means	2.116988	10033.3	0.60
	hierarchical agglomerative	1.809972	9868.8	0.60

Nonparametric clustering analysis of MD simulation of Est2 and Est3 was carried out for the determination of the most two populated clusters by the hierarchical agglomerative algorithm. The ratios of the two most populated clusters were determined as 29.6–19.4% for Est2 (Figure S5a) and 31.5–14.4% (Figure S5b). The residues surrounding the ligand within 4 Å were investigated in the conformation of the most populated first two clusters in Est2 (Figure S5b,c) and Est3 (Figure S6b,c). According to this analysis, for Est2, the common residues surrounding the ligand within 4 Å in the most populated two clusters in Est2 were F25, P118, K122, T126, M127, G130, L167, M195, and I196 and these residues were accepted as the ligand-binding sites. On the other hand, the common residues around the ligand within 4 Å in the most populated two clusters in Est3 were found as F25, P66, W69, L95, F99, L167, and L170, and the residues were accepted as the ligand-binding sites.

**Figure 6:**
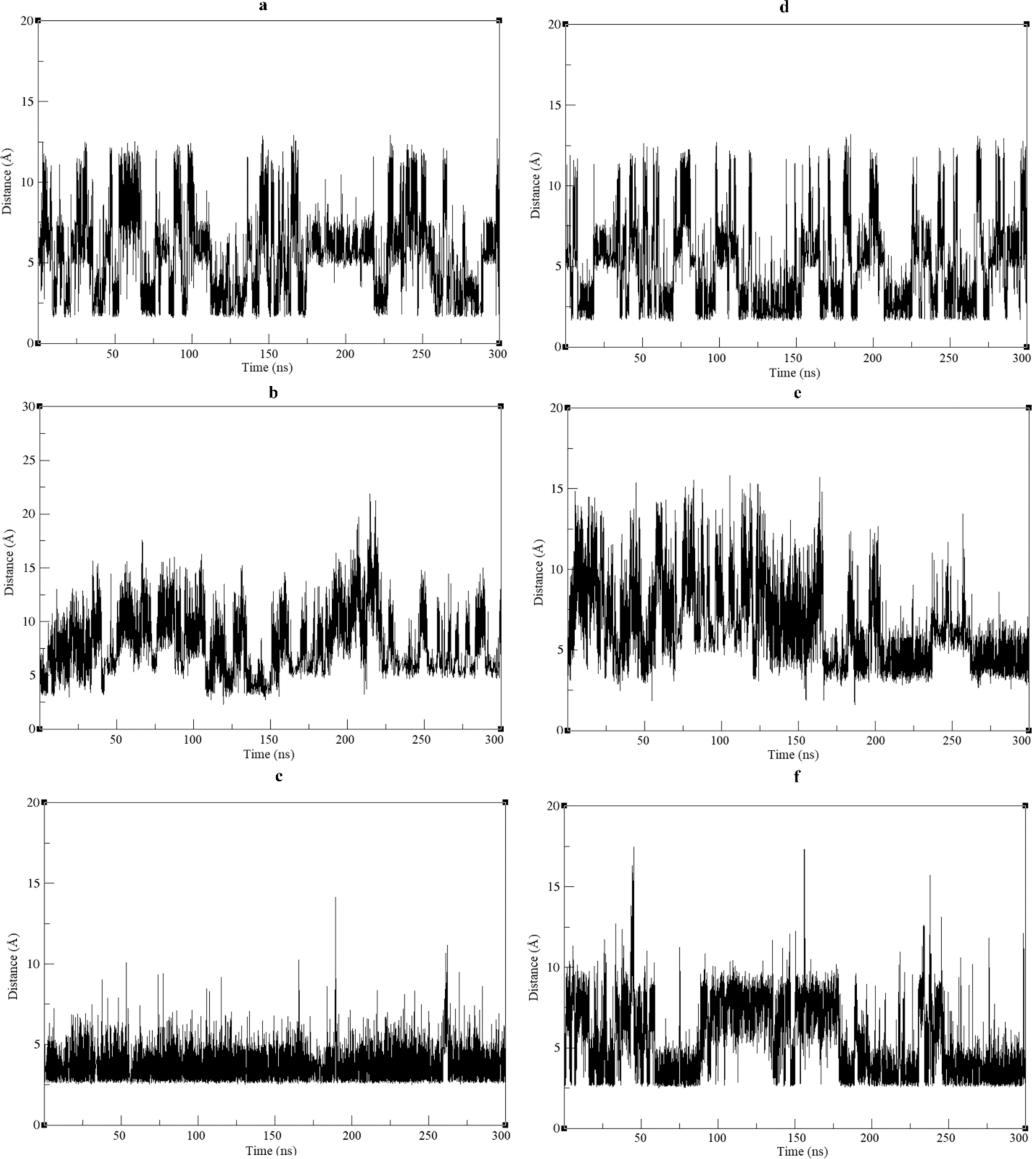
Distance analysis of E40–R37, E150–R136, and E243–R17 in Est2 (a–c) and Est3 (d–f). (a, d) E40 OE1–R37 HH11; (b, e) E150 OE2–R136 HH11; and (c, f) E243 OE1–R17 NH1.

The dynamics analysis of the distance occurring between some specific residues, which can potentially make a salt bridge, was investigated in Est2 and Est3. This analysis showed that the distance of E40 OE1 (double-bonded oxygen atom to the carbon atom in the carboxyl group of the side chain) to R37 HH11 (hydrogen atom bound to the double-bonded nitrogen atoms in the guanidinium group of its side chain), E150 OE2 (single-bonded oxygen atom to the carbon atom in the carboxyl group of the side chain) to R136 HH11, and E243 OE1 to R17 NH1 (double-bonded nitrogen atoms in the guanidinium group of its side chain) exhibited similarity between Est2 and Est3 throughout the MD simulation. The distance could frequently reduce below 4 Å, which was a distance to make a salt bridge between positively charged and negatively charged residues in Est2 and Est3 (Figure 6).

## 4 Discussion

In this work, two thermostable esterase amino acid sequences of *Geobacillus* species isolated from Balçova geothermal water and experimentally characterized by Tekedar and Şanlı-Mohamed (2011)^17^ were *in silico* characterized at sequence and structure level, compared to the esterase from *G. stearothermophilus* (Est30). The amino acid sequences of two thermostable esterases were retrieved from NCBI by using their accession numbers FN597622 and FN597623 for Est2 and Est3, respectively.

Biophysicochemical aspects, such as theoretical *T*_m_, aliphatic index, and theoretical pI of the Est2, Est3, and Est30, were found in the interval of 55–65 °C, 82–83, and 5.0–5.25, respectively, and were similar to each other (Table 1). It is known that a higher aliphatic index is an indicator of a higher thermostability.^53^ In line with this, these three enzymes share a thermophilic character. Accordingly, Est2 and Est3 experimentally had optimal activity at 65 °C,^17^ whereas the optimal temperature of Est30 was found as 70 °C,^47^ supporting the similarity of the aliphatic index of the three esterases.

Also, lipolytic enzymes including esterases are widely recognized for featuring catalytic triad residues (Ser-Asp/Glu-His), which include the serine amino acid within their active sites, resembling serine proteases. These enzymes operate at the interface between oil and water.^54^ In alignment with this, Est30 also possesses three catalytic cleft amino acids (S93, D192, and H222), as revealed by X-ray crystallography deposited in the RCSB PDB databank under accession number 1TQH.^48^ In this work, multiple sequence alignment (MSA) analysis showed that the corresponding catalytic triad residues for Est2 and Est3 were S94 in the β4–α3 loop, D193 in the β6–α7 loop, and H223 in the β7–α8 loop (Figure 1). Furthermore, Family XIII esterases, such as Est30, bear the highly conserved GLSLG pentapeptide around the catalytic serine, with D and H serving as the catalytic triad residues.^45,47,48^ Consistent with this, the sequence alignment analysis revealed that two thermostable esterases shared a consensus GLSLG sequence (Figure 1).

In this study, structural assessment of Est2 and Est3 showed that two thermostable esterases possessed a three-helix cap domain and an α/β-hydrolase fold domain (Figure 2). Similarly, *Ea*Est2 displayed an overall structure characterized by seven β-strands and 10 α-helices, adopting the classical α–β hydrolase fold in the central domain. This core domain featured a twisted β sheet surrounded by α-helices and included a cap domain. The substrate-binding pocket, notably, was concealed by the cap domain, giving it an elongated and narrow configuration.^55^ In contrast to the lid domain found in numerous lipases, the cap domain did not interfere with the catalytic function of the enzyme. Comparable instances of a small cap were also identified in the structures of Est30 from *G. stearothermophilus*, CEGk from *Geobacillus kaustophilus*, Est0796 from *Lactobacillus plantarum*, EstOF4 from *Bacillus pseudofirmus* OF4, and EstA from *Streptomyces lividans* TK24.^49,56−59^ Certainly, the active region and the site for substrate binding were positioned at the interface of the domains, where the presence of the small cap contributes to the enzyme’s substrate specificities. This cap domain structure is characterized as a small cap domain in the literature. Certainly, the active region and substrate-binding site were positioned at the interface of the domain, where the small cap contributes to the enzyme’s substrate specificities.^60^

BANΔIT analysis showed that regions 8, 9, 10, and 11, which were much more rigid parts than that of Est30, indicating thermostability, were placed in the α4 helix, α4–α5 loop, α5 helix, and α5–α6 loop, respectively, and they were located at the cap domain (Figure 3). Gall et al. (2014) showed that altering the cap domain of *Bacillus subtilis* esterase (BsubE) through the introduction of 10 amino acids with enhanced rigidity, distinct from the *G. stearothermophilus* esterase (BsteE), resulted in a 4 °C increase in the melting temperature compared to the native BsubE enzyme.^61^ In addition, salt bridge analysis indicated that Est2 and Est3 had a higher number of salt bridges at the overall structure and cap domain, compared to Est30 (Figure 4). The idea of electrostatic interaction or salt bridges is a fundamental factor in protein stabilization, with thermostable proteins displaying a greater frequency of salt bridges in comparison to proteins adapted to moderate temperatures.^62^ In the literature, the residual activity of Est2 and Est3 was determined as 90% upon 1 h of incubation at 65 °C;^17^ however, the activity of Est30 retained about 85 and 75% at 60 and 70 °C, respectively, after 1 h.^47^ This indicated that Est2 and Est3 demonstrated a relatively more thermostable profile when compared to Est30, and this supported the salt bridge and *B*′-factor findings in the present work. Thus, this work proposed that higher rigidity and more salt bridges of the cap domain in Est2 and Est3 might contribute to greater thermal stability relative to Est30.

In this work, ligand-binding site residues were found as F25, P118, K122, T126, M127, G130, L167, M195, and I196 in Est2 (Figure S5b,c). DeepMSA2 analysis indicated that F25, P118, L167, M195, and I196 were conserved in a significant number of sequences. However, K122, T126, M127, and G130 were not conserved in almost all of the sequences (Figure S2). Nonconserved residues T126, M127, and G130 were located at helix α4, which was a region of the cap domain (Figure 2). Also, the ligand-binding site residues in Est3 were found as F25, P66, W69, L95, F99, L167, and L170 (Figure S6b,c). Among these, P66 and L170, which were located at helix α2 (Figure 2), were not conserved in almost all of the sequences, whereas F25, W69, L95, F99, and L167 were conserved in a great number of homologous esterases (Figure S2).

In addition, Tekedar and Şanlı-Mohamed (2011) have reported that the relative activity of Est3 was higher at pH 6–9 and 9 and at high temperatures (70–80 °C) than Est2. The same work showed that the catalytic efficiency of Est3 was greater than that of Est2.^17^ Thus, this work focuses on the amino acid differences between Est2 and Est3. Accordingly, Est2 possessed a proline at position 6; however, threonine was present in Est3 at the same position (Figure 1). This position was located near region 1 of the N-terminal region in the RMSF graph, as shown in Figure 5b. In the most populated cluster of Est3, it was noted that T6 was less than 4 Å away from S29. However, P6 in the most populated cluster of Est2 was not as close to any amino acid between positions 20 and 30 (data not shown). This indicated that T6 in Est3, unlike in Est2, had the potential to form polar interactions, thus explaining the amino acid fluctuation in this region. Also, at position 110, Est2 and Est3 possessed glutamine and glutamic acid, respectively (Figure 1). This position was found near region 2 of the cap domain in the RMSF graph, as shown in Figure 5b. When examining the amino acids within 4 Å of Q110 and E110, it was observed that Est2, unlike Est3, contained the amino acids L245, D246, and W247. The MSA results showed that these three amino acids were present at the end of Est2, but not found in Est3. The potential interactions between Q110 and these three amino acids in Est2 might stabilize region 2 and lead to less fluctuation, compared to Est3. Interestingly, these extra residues found in Est2 were another difference between Est2 and Est3, and they were placed near region 3 of the C-terminal region, including catalytic residue H223, in the RMSF graph, as shown in Figure 5b. This work suggested that the fluctuation of the residues in the N-terminal region including F25, cap domain, and C-terminal region including H223 might affect the catalytic efficiency of the thermostable esterases.

The distance analysis indicated that the distance profiles of E40 OE1 to R37 HH11, E150 OE2 to R136 HH11, and E243 OE1 to R17 NH1 were very similar in Est2 and Est3 along the MD simulation duration (Figure 6). The similarity between Est2 and Est3 in terms of salt bridge dynamics was consistent with the total salt bridge analysis (Figure 4) and supported its conclusion.

## 5 Conclusions

This study presents an in silico characterization of the two biochemically characterized thermostable esterases (Est2 and Est3) of *Geobacillus* strains isolated from the Balçova geothermal area in İzmir, Turkey. The aliphatic indices and theoretical *T*_m_ values of the esterases ranged approximately between 82–83 and 55–65 °C, respectively, indicating their thermostability. The molecular phylogeny results revealed that Est2 and Est3 were classified within Family XIII, where the Geobacillus esterases were found. DeepMSA2 analysis indicated all Est2 and Est3 possessed 12 residues in the core domain (L39, D50, G53, G55, S57 G92, S94, G96, P108, P184, D193, and H223), as conserved in all of the homologous sequences. Different from the homologous sequences, four core domain residues (G104 in α3, I109 in α3–β5 loop, and F186-A190 in β6) were found in Est2 and Est3. BANΔIT analysis showed that Est2 and Est3 possessed significantly more rigid cap domains relative to Est30, indicating a higher level of thermostability. The cap domain of the esterases was stabilized by the salt bridge network, suggesting that this could contribute to the thermostability of the enzymes. MD simulation results showed that Est3 had a higher fluctuation in the N-terminal region, in the cap domain, and C-terminal region including H223, suggesting that these regions might influence the catalysis of Est2 and Est3. The common residues in ligand-binding sites of Est2 and Est3 were determined as F25 and L167. RMSF analysis showed that region 1 including F25 in the β2–α1 loop of Est3 had greater fluctuation than Est2. This research could provide direction for investigations aimed at enhancing the thermostability and catalytic efficiency, a crucial industrial characteristic of esterases, through targeted modification of certain amino acids within the N-terminal region, the cap domain, and the C-terminal region using rational protein engineering techniques.

## Supporting Information Available

Phylogenetic tree; deep multiple sequence alignment; cross-validation of enzyme models; and clustering analysis (PDF)
