# Supplementary material for: Exploring the Structural Insights of Thermostable Geobacillus esterases by Computational Characterization
Source: ACS Omega. 2024 Jul 22;9(30):32931–41. doi: 10.1021/acsomega.4c03818 (PMC11292637; doi:10.1021/acsomega.4c03818)
Supplement: Supplementary file 1 — ao4c03818_si_001.pdf [file ao4c03818_si_001.pdf]

Supporting Information

Exploring the structural insights of thermostable *Geobacillus* esterases by computational characterization

Yusuf Sürmeli[1*], Naciye Durmuş[2] and Gülşah Şanlı-Mohamed[3*]

1. Department of Agricultural Biotechnology, Tekirdağ Namık Kemal University, 59030, Tekirdağ, Turkey
2. Department of Molecular Biology and Genetics, İstanbul Technical University, 34485, İstanbul, Turkey
3. Department of Chemistry, İzmir Institute of Technology, 35430, İzmir, Turkey

***Corresponding Authors**

Assoc. Prof. Dr. Yusuf Sürmeli

Tekirdağ Namık Kemal University, Faculty of Agriculture,

Department of Agricultural Biotechnology, Süleymanpaşa, Tekirdağ, Turkey

Phone: +90 2822502249; Fax: +90 2822509929

E-mail: ysurmeli@nku.edu.tr, ysurmeli1985@gmail.com

Prof. Dr. Gülşah Şanlı-Mohamed

Department of Chemistry, Science Faculty, Izmir Institute of Technology, Urla, Izmir, Turkey

Phone: +90 2327507515; Fax: +90 2327507509

E-mail: gulsahsanli@iyte.edu.tr, gulsahsanli@hotmail.com

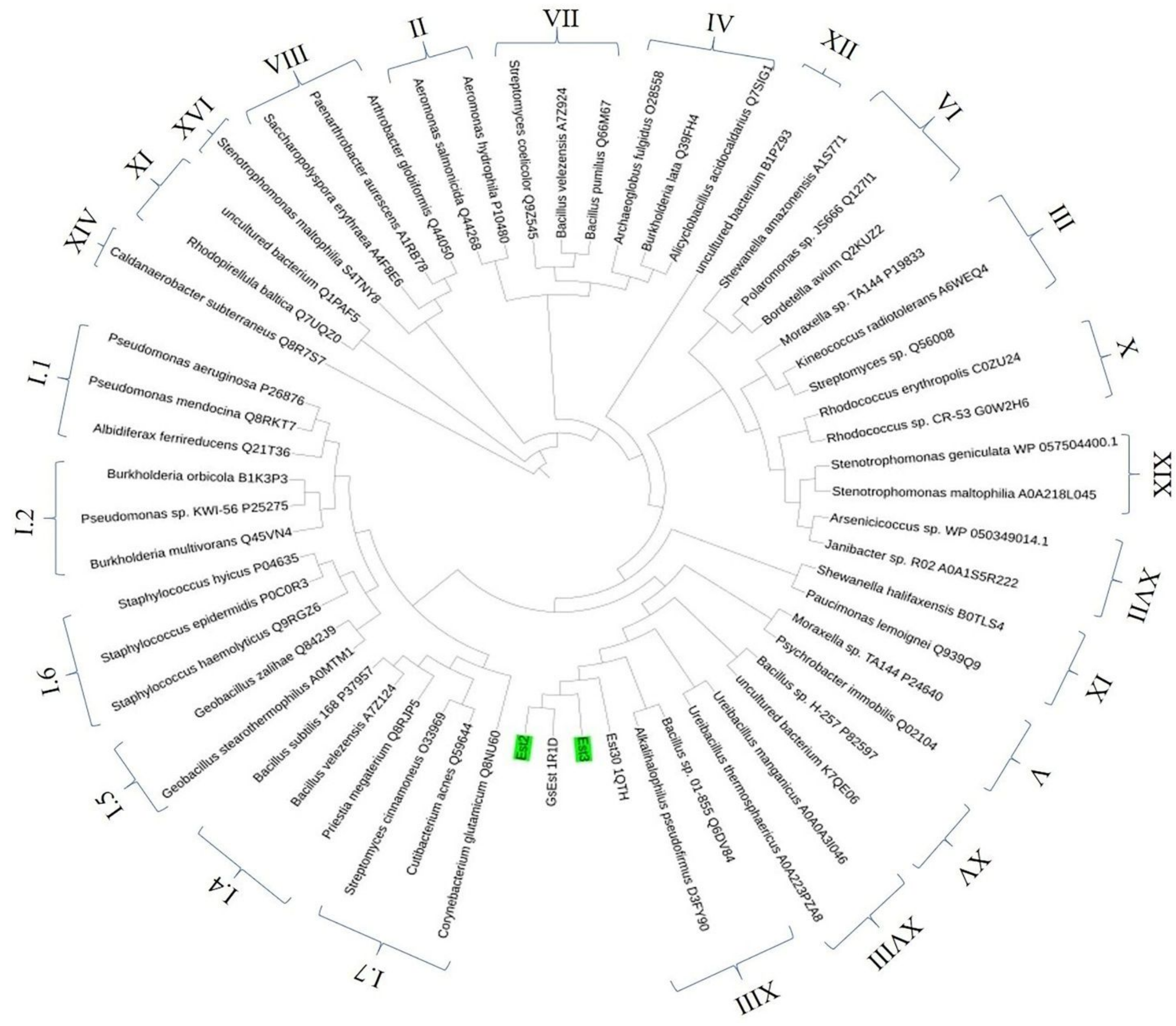

Figure S1. The phylogenetics relationships of the amino acid sequences of Est2 and Est3 with the other esterases lipases of 57 protein sequences from different bacterial sources. The UniProt, GeneBank or PDB accession numbers, and the name of the microorganisms were supplied.

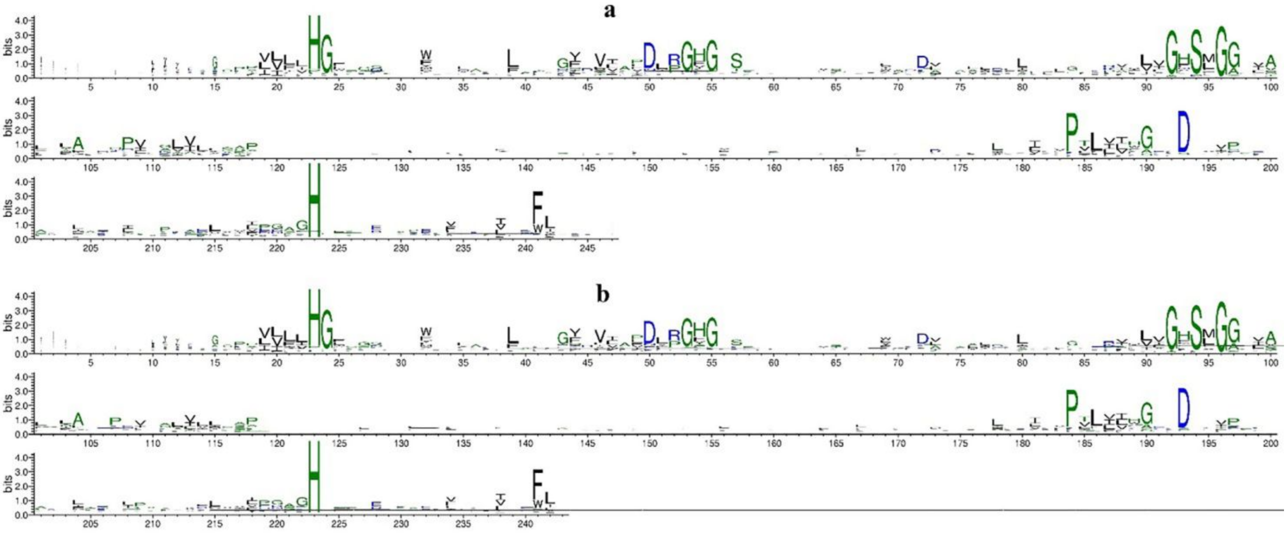

Figure S2. Deep multiple sequence alignment (DeepMSA2) of the **a)** Est2 and **b)** Est3 to determine the conserved and non-conserved residues, compared with the homologous esterases in the Uniclust30, Uniref90, BFD, MGnify and IMG/M databases.

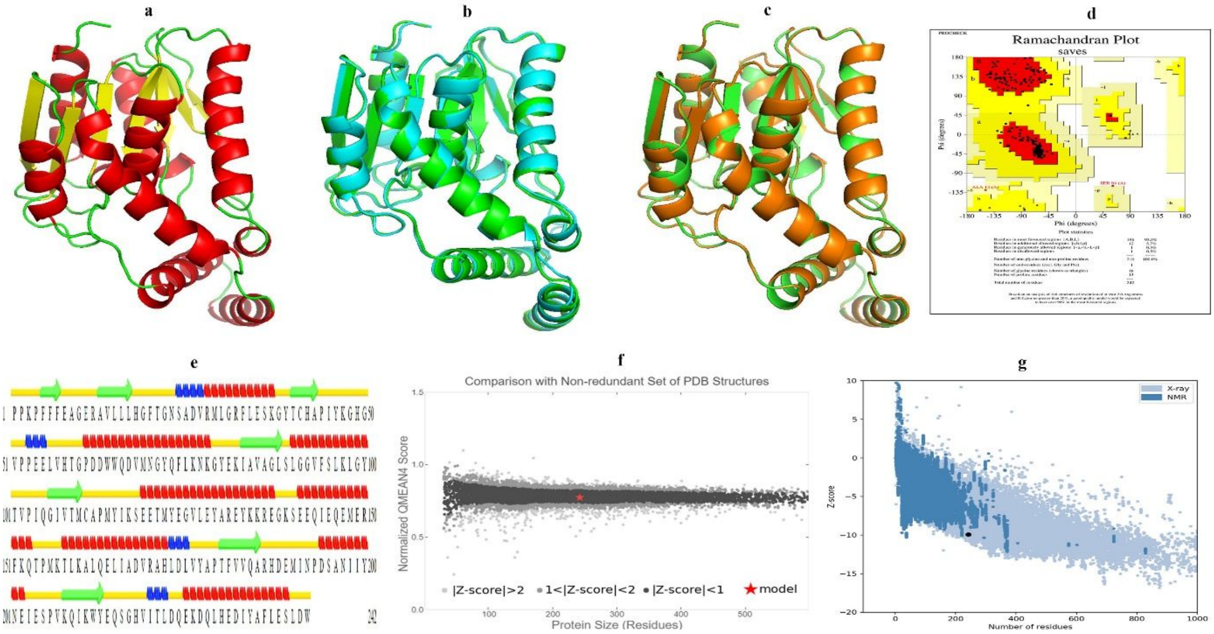

Figure S3. Cross-validation analyses of homology model of Est2. **a)** The 3D predicted structure obtained from SWISS-MODEL. **b)** The structural alignment between the homology model (green) and alphafold model (cyan) displayed by PyMOL. **c)** The structural alignment between the homology model (green) and the best template (orange) displayed by PyMOL. **d)** Ramachandran plot of the homology model, built by ProCheck. **e)** The prediction of

secondary structure of the homology model by Stride. **f)** QMEAN prediction of homology model acquired by SWISS-MODEL. **g)** Overall quality of the homology model evaluated by the ProSA server.

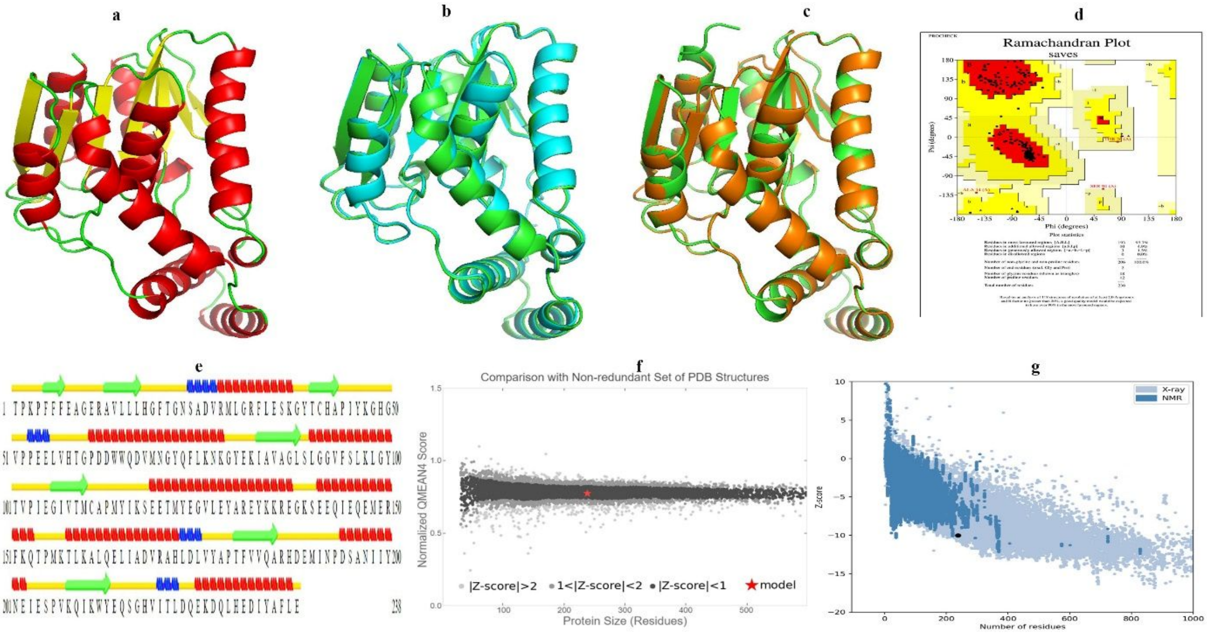

Figure S4. Cross-validation analyses of homology model of Est3. **a)** The 3D predicted structure obtained from SWISS-MODEL. **b)** The structural alignment between the homology model (green) and alphafold model (cyan) displayed by PyMOL. **c)** The structural alignment between the homology model (green) and the best template (orange) displayed by PyMOL. **d)** Ramachandran plot of the homology model, built by ProCheck. **e)** The prediction of secondary structure of the homology model by Stride. **f)** QMEAN prediction of homology model acquired by SWISS-MODEL. **g)** Overall quality of the homology model evaluated by the ProSA server.

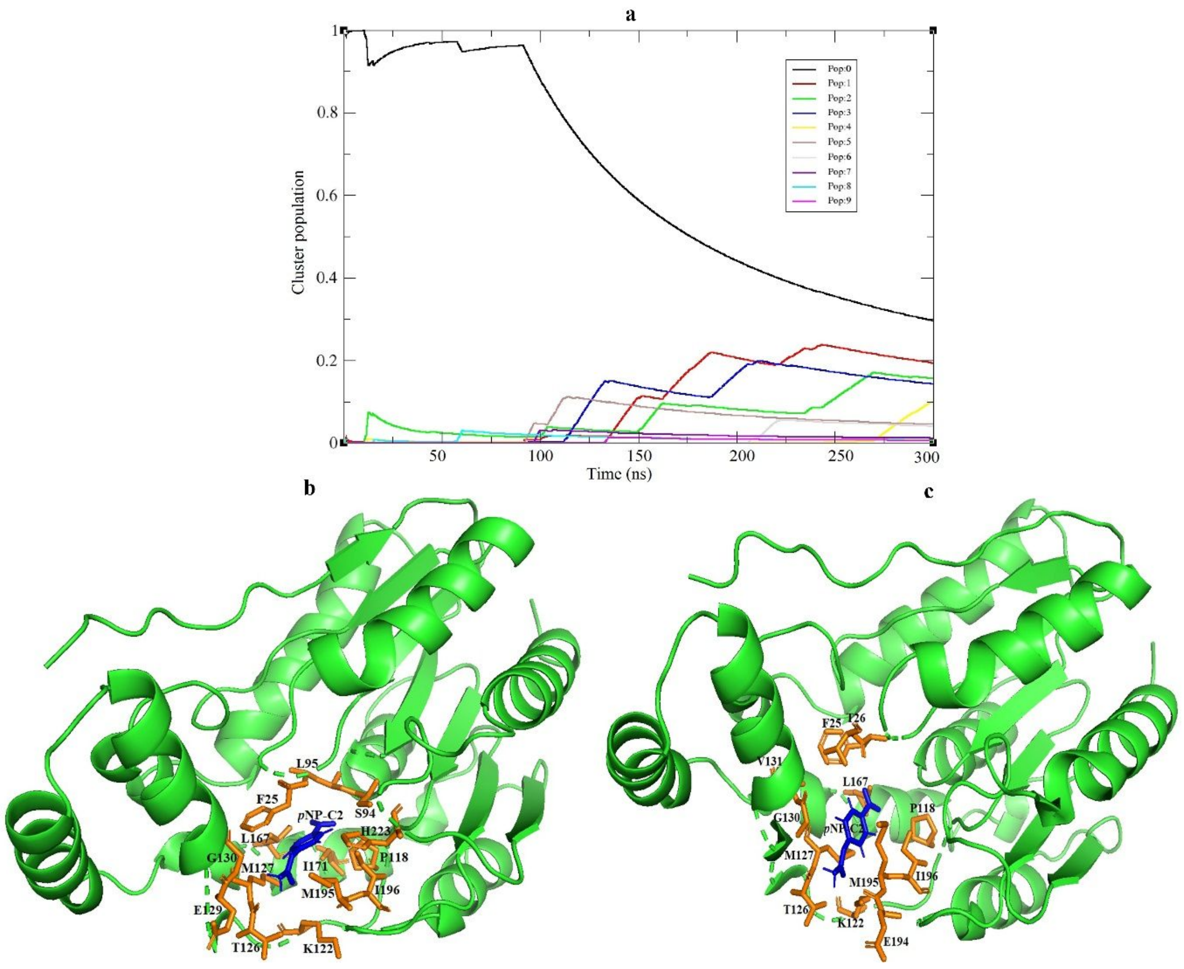

Figure S5. Clustering analysis of the Est2 by hierarchical agglomerative algorithm. **a)** the changes of cluster populations in time, **b)** the conformation of the first most populated cluster and the residues surrounded ligand within 4 Å, **c)** the conformation of the second most populated cluster and the residues surrounded ligand within 4 Å

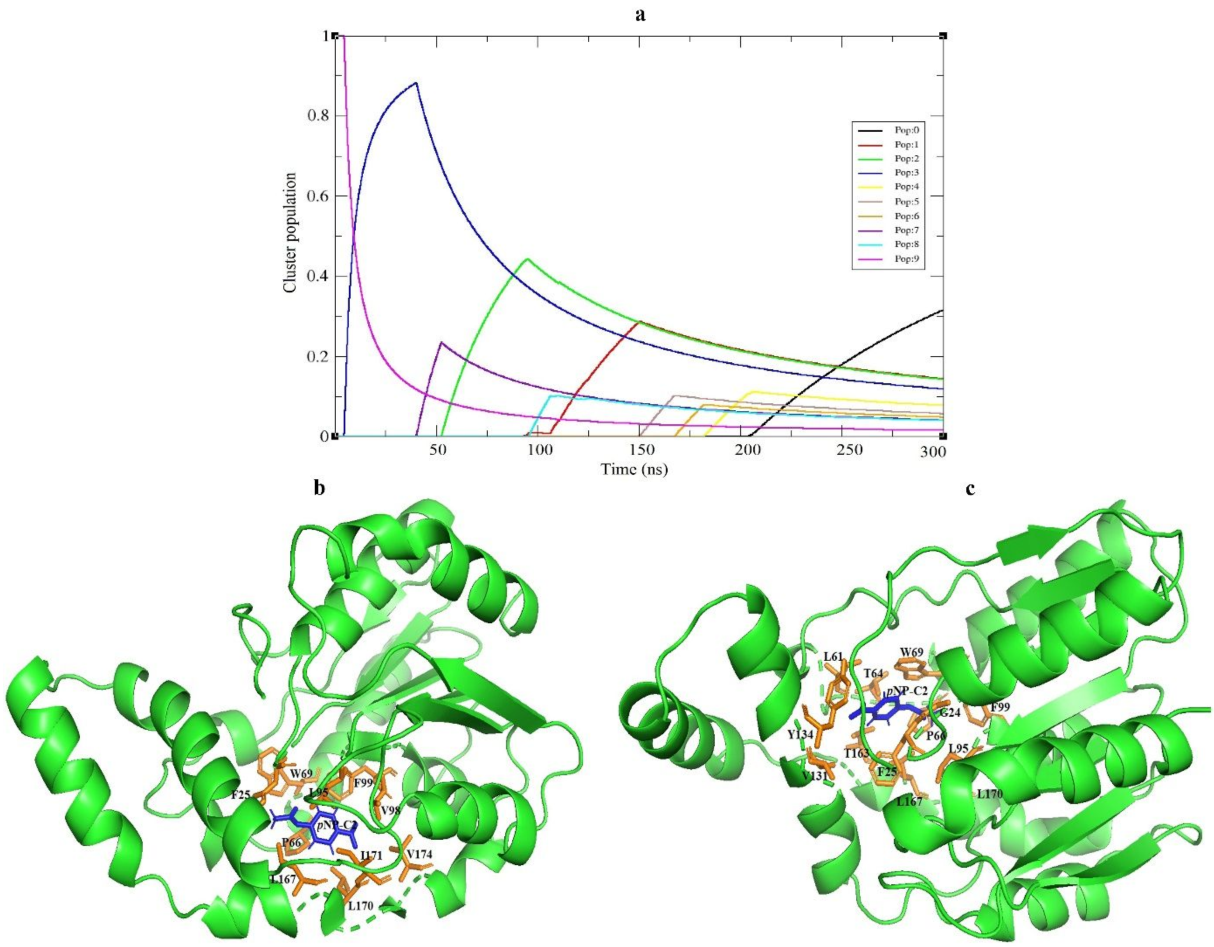

Figure S6. Clustering analysis of the Est3 by hierarchical agglomerative algorithm. **a)** the changes of cluster populations in time, **b)** the conformation of the first most populated cluster and the residues surrounded ligand within 4 Å, **c)** the conformation of the second most populated cluster and the residues surrounded ligand within 4 Å
